# High Thyroglobulin Antibody Following Intravenous Immunoglobulin Therapy in Athyreotic Differentiated Thyroid Cancer Patients

**DOI:** 10.7759/cureus.32103

**Published:** 2022-12-01

**Authors:** Kheng Joe Lau, Gurunanthan Palani, Claudio Brunstein, Lynn A Burmeister

**Affiliations:** 1 Endocrinology, Diabetes and Metabolism, University of Minnesota School of Medicine, Minneapolis, USA; 2 Hematology and Medical Oncology, University of Minnesota School of Medicine, Minneapolis, USA

**Keywords:** false positive, thyroglobulin antibody, thyroid cancer, intravenous immunoglobulin therapy, intravenous immunoglobulin (ivig), intravenous immunoglobulin

## Abstract

American Thyroid Association guidelines recommend to follow athyreotic differentiated thyroid cancer patients with measurement of serum thyroglobulin and thyroglobulin antibody as tumor markers. The guidelines recommend that rising thyroglobulin or thyroglobulin antibody should prompt additional investigations and potentially additional therapies.

Two patients with differentiated thyroid cancer who also received intravenous immunoglobulin are presented. Their cancer history, serial thyroglobulin and thyroglobulin antibody measurements and imaging findings relative to the time course of intravenous immunoglobulin treatment are illustrated. Acute rise in thyroglobulin antibody led to further imaging which did not show cancer progression. Additional history documented an intravenous immunoglobulin treatment exposure had occurred within the past one to two months before the increased thyroglobulin antibody measurement. Follow-up serial thyroglobulin antibody levels declined over time after the intravenous immunoglobulin exposure.

Intravenous immunoglobulin, which is a pooled human serum product, contains thyroglobulin antibody. Commercially available thyroglobulin antibody assays may detect the thyroglobulin antibody contained within the administered intravenous immunoglobulin, leading to alarm and further imaging to exclude progressive malignancy. Thyroglobulin antibody rise and fall can be demonstrated in relationship to intravenous immunoglobulin time of administration. Thyroglobulin antibody is higher at time-points sooner than at later time-points following intravenous immunoglobulin treatments.

Intravenous immunoglobulin may be a benign source of transiently high thyroglobulin antibody measured in the follow-up of differentiated thyroid cancer patients. Repeat thyroglobulin and thyroglobulin antibody testing one to two months following a higher level in a patient treated with intravenous immunoglobulin may avoid unnecessary imaging to look for progressive malignancy.

## Introduction

American Thyroid Association guidelines recommend to follow athyreotic differentiated thyroid cancer patients with measurement of serum thyroglobulin and thyroglobulin antibody as tumor markers [1]. The guidelines recommend that rising thyroglobulin or thyroglobulin antibody should prompt additional cancer investigations and potentially additional therapies [1]. We present two patients demonstrating rise in thyroglobulin antibody which was not due to cancer progression, but rather was due to intravenous immunoglobulin administration. This article was previously presented as an e-poster at the 2020 American Association of Clinical Endocrinologists 29th Annual Scientific and Clinical Congress meeting EMBRAACE May 2020.

## Case presentation

Case one

A 67-year-old woman underwent total thyroidectomy in 2016. Pathology showed 0.8 cm oncocytic variant papillary thyroid carcinoma with microscopic extra-thyroid extension and four benign lymph nodes (AJCC 8 TNM stage 1). Whole-body radioiodine scan in 2016 showed no abnormal uptake. She was not treated with radioiodine.

Her past medical history included autologous hematopoietic cell transplant for treatment of chronic lymphocytic leukemia with transformation to diffuse large B-cell lymphoma, resulting in complete remission. Secondary persistent hypoglobulinemia was being treated with intravenous immunoglobulin infusion every three to six months.

**Figure 1:**
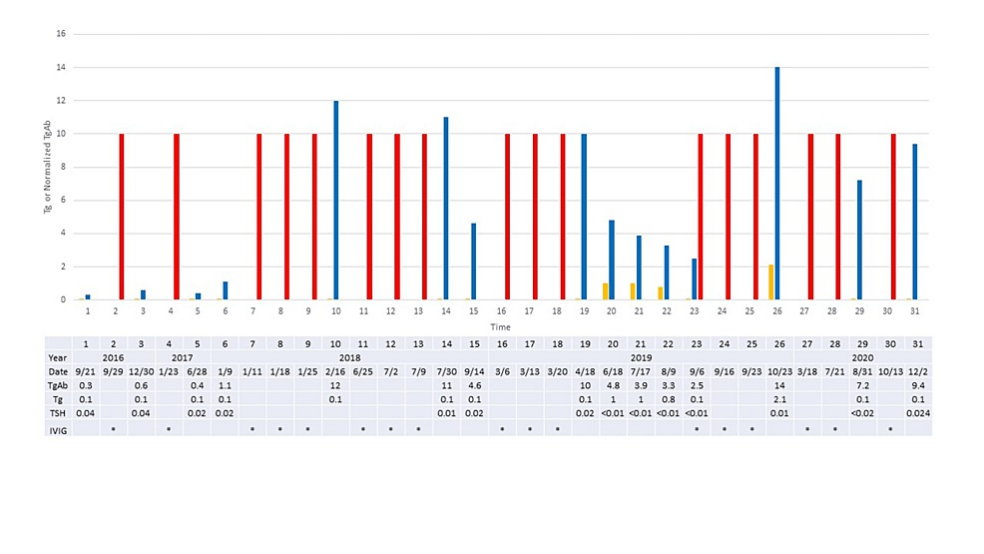
Case 1 thyroglobulin and normalized thyroglobulin antibody Serial measurements of thyroglobulin (Tg) and normalized thyroglobulin antibody (TgAb) in case 1. Normalization of TgAb was relative to the reference assay upper normal level to compensate for differences in assays that were used. Blue bars denote normalized TgAb, gold bars denote Tg levels (mcg/L), red bars show times of intravenous immunoglobulin (IVIG) administration. The x-axis shows consecutive times. Figure table shows Tg in mcg/L, normalized TgAb and the date of testing. IVIG administration is denoted with * on the figure table. TgAb was performed by four different laboratories including HPMG Laboratories (reference range 0-3.9 IU/mL), ARUP Laboratories (reference range 0-4 IU/mL Beckman Coulter Access DxI), Mayo Clinic laboratories (reference range < 1.8 IU/mL Beckman Coulter Unicel DXI 800), and USC Endocrine laboratory.

Follow-up serial thyroglobulin, thyroglobulin antibody and thyroid-stimulating hormone (TSH) levels are shown in Figure 1, initially raising concern when the thyroglobulin antibody rose over 10-fold from 4.2 (normalized value 1.1) to 46.3 mU/L (normalized value 12) in 2/2018. 123I total body scan with single-photon emission computerized tomography showed thyroid bed uptake, unchanged compared with 2016 and no distant metastasis. Computerized tomography of neck, chest, abdomen and pelvis in 6/2018 and 7/2019 did not suggest metastatic presence or progression compared with 2014 images. Neck ultrasound (6/2018) showed no adenopathy. The record documented multiple intravenous immunoglobulin infusions over time with instability of serial thyroglobulin antibody levels (Figure 1).

**Figure 2:**
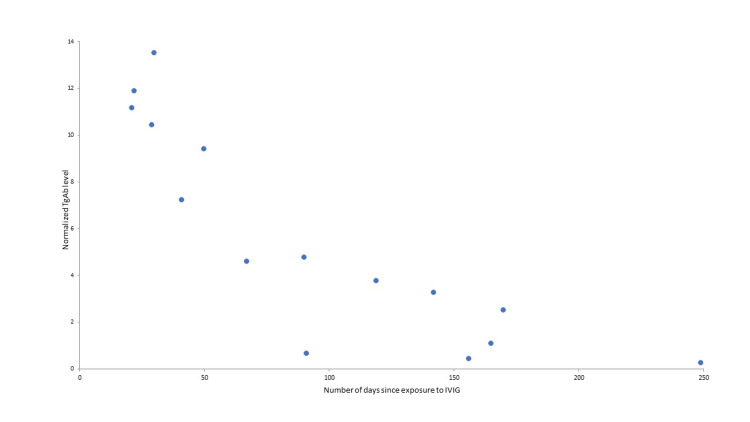
Case 1 thyroglobulin antibody versus days since intravenous immunoglobulin Normalized thyroglobulin antibody (TgAb) levels versus days since intravenous immunoglobulin (IVIG) exposure for case 1. Normalization of TgAb was relative to the reference assay upper normal level to compensate for differences in assays that were used.

In contrast, Figure 2 demonstrates the inverse relationship between the thyroglobulin antibody level and the days following the last dose of intravenous immunoglobulin, in support of the intravenous immunoglobulin as the source of the thyroglobulin antibody.

Case two

**Figure 3:**
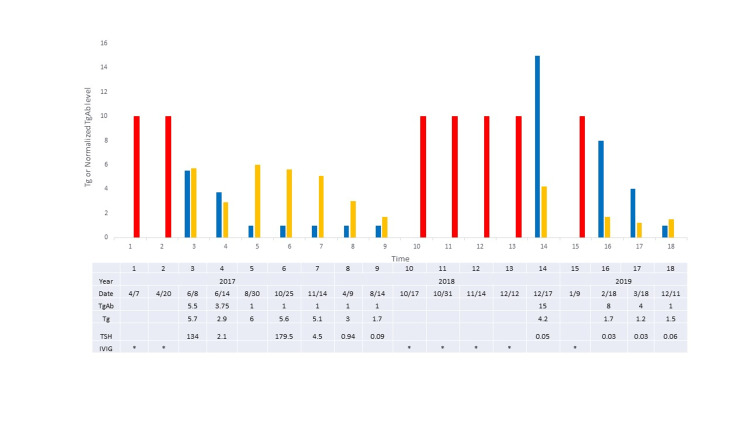
Case 2 thyroglobulin and normalized thyroglobulin antibody. Serial measurements for thyroglobulin (Tg) and normalized thyroglobulin antibody (TgAb) in case 2. Blue bars denote normalized TgAb, gold bars denote Tg levels (mcg/L), red bars show times of intravenous immunoglobulin (IVIG) administration. The x-axis shows consecutive times. Figure tables show Tg in mcg/L, normalized TgAb and the date of testing. IVIG administration is denoted with * on the figure tables. Both Tg and TgAb were performed exclusively at USC Endocrine laboratory. Tg was measured by radioimmunoassay when TgAb was above normal and by immunometric assay when TgAb was normal. The reference range for TgAb is < 0.4 U/mL.

A 75-year-old man with a history of chronic inflammatory demyelinating polyneuropathy was treated in 2017 with total thyroidectomy, central and lateral neck dissection. Pathology showed multifocal papillary thyroid carcinoma, largest nodule 0.5 cm, with 14/31 positive lymph nodes with extra-nodal extension. Chest computerized tomography showed multiple pulmonary nodules measuring up to 3 mm in size. He received 149 mCi I-131, unfortunately days following an unrecognized iodinated computerized tomography contrast exposure in another health system. Second dose 197 mci I-131 was given five months later. Post-therapy scans did not show uptake in the lung nodules on either treatment. Fluorodeoxyglucose-positron emission tomography was negative. Thyroglobulin and thyroglobulin antibody progressively fell over the next year following treatment. Specifically, initial TSH-stimulated 5/18/17 thyroglobulin 5.7 mcg/L, thyroglobulin antibody 2.2 mU/L (normalized thyroglobulin antibody 5.5) fell to 5.6 mcg/L and < 0.4 mU/L (normalized thyroglobulin antibody 1) prior to the second dose of radioiodine. TSH-suppressed thyroglobulin continued to fall to 1.7 mcg/L with thyroglobulin antibody < 0.4 mU/L (normalized thyroglobulin antibody 1) by August 2018. 11/18 chest computerized tomography remained stable with 4 mm lung nodules. On 12/17/2018 the TSH-suppressed thyroglobulin was higher at 4.2 mcg/L with higher thyroglobulin antibody 6 mU/L (normalized thyroglobulin antibody 15), raising concern for disease progression. Serial thyroglobulin and thyroglobulin antibody levels are shown in Figure 3.

**Figure 4:**
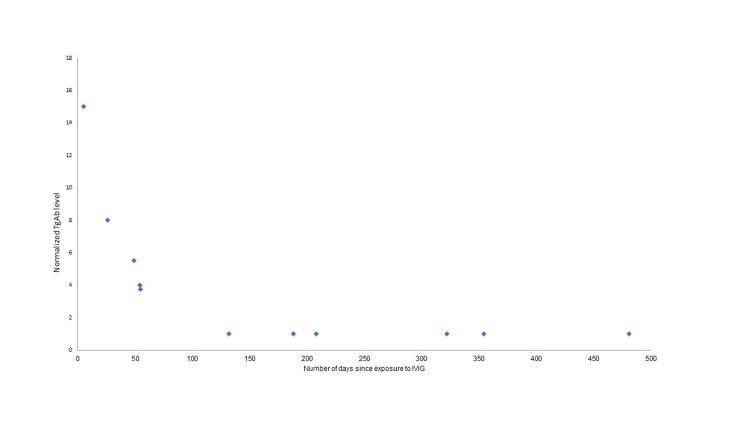
Case 2 thyroglobulin antibody versus days since intravenous immunoglobulin (IVIG) Normalization of thyroglobulin antibody (TgAb) was relative to the reference assay upper normal level to compensate for differences in assays that were used.

Neck ultrasound did not show abnormality. As further imaging was considered, the patient disclosed that intravenous immunoglobulin treatment had been given 33 days prior to the lab measurement in another health system for treatment of chronic inflammatory demyelinating polyneuropathy. The next round of intravenous immunoglobulin was followed by thyroglobulin 1.7 mcg/L and thyroglobulin antibody 3.2 mU/L (normalized thyroglobulin antibody 8). With discontinuation of intravenous immunoglobulin the thyroglobulin and thyroglobulin antibody returned to baseline levels of 1.5 mcg/L and < 0.4 mU/L (normalized thyroglobulin antibody 1) and remained there off intravenous immunoglobulin for another year. Figure 4 demonstrates the inverse relationship between the thyroglobulin antibody level and the days following the last dose of intravenous immunoglobulin for case 2, in support of the intravenous immunoglobulin as the source of the thyroglobulin antibody.

## Discussion

Two thyroid cancer patients, who were also receiving intravenous immunoglobulin, demonstrated intermittently higher thyroglobulin antibody that did not correlate with cancer progression or recurrence. Rather, the thyroglobulin antibody showed recurrent rise and fall with each intravenous immunoglobulin treatment (Figures 1, 3), with higher levels closer in time to the exposure and lower levels longer after the exposure (Figures 2, 4). At least two different intravenous immunoglobulin brands and four different thyroglobulin antibody assays, with different reference ranges [2], were used in these patients, but all showed the same pattern of thyroglobulin antibody rise following intravenous immunoglobulin. Other causes of falsely high thyroglobulin antibody, including heterophile antibody or biotin ingestion, were not thought to be present [1,3]. The observed changes in thyroglobulin antibody were most consistent with measurement of thyroglobulin antibody present in the intravenous immunoglobulin. 

Intravenous immunoglobulin is known to cause some laboratory assay interferences but has not been widely appreciated as a cause of elevated thyroglobulin antibody results. We are aware of only three other reported cases of increased thyroglobulin antibody following immunoglobulin administration [3-5].

Intravenous immunoglobulin, used for treatment of specific immunodeficiency or autoimmune syndromes, is made from pooled serum of 1000-100,000 healthy human donors. It contains the polyclonal natural antibody immunoglobulin classes normally present in plasma (consisting mainly of immunoglobulin G), as well as other plasma factors [6]. The half-life of intravenous immunoglobulin is similar to blood immunoglobulin, but the half-life can vary depending on the underlying treatment condition and intravenous immunoglobulin preparation [6].

Thyroglobulin antibody is present in 10.4%-38% of healthy humans [7,8] as well as in up to 90% with autoimmune thyroid disease [9,10]. Like other natural autoantibodies, thyroglobulin antibody has been demonstrated in pooled normal human serum [11]. Likewise, intravenous immunoglobulin, being a pooled serum product, contains thyroglobulin antibody [3,12-15], reflecting the integrated levels from all donor sources with their different thyroglobulin antibody epitope specificities. Thus, the specificity of the thyroglobulin antibody in different intravenous immunoglobulin batches may differ such that successive intravenous immunoglobulin treatments may influence a patient’s thyroglobulin and thyroglobulin antibody result to different degrees. Thyroglobulin antibody is not the only laboratory test that may be affected following intravenous immunoglobulin. False serologic laboratory test result due to intravenous immunoglobulin has been described for infectious agents, red cell and other autoantibodies [16,17].

When thyroglobulin antibody is present in thyroid cancer patients who have also received intravenous immunoglobulin, short-term serial measurements may demonstrate clearance of thyroglobulin antibody. Clearance within seven to 21 days has been reported in healthy humans and within 33-36 days in immunodeficient patients [6]. Plotting of normalized thyroglobulin antibody levels over time from intravenous immunoglobulin exposure suggested more prolonged thyroglobulin antibody clearance following intravenous immunoglobulin in case one, who was being treated for immunodeficiency, than that of case two who did not have immunodeficiency. In both cases, the clearance kinetics appear to demonstrate an initial distribution phase lasting one to two months and a more prolonged terminal phase.

## Conclusions

Proper interpretation of laboratory tests often requires context. Rise and fall of thyroglobulin antibody levels can be demonstrated after immunoglobulin treatment in the follow-up of athyreotic patients with differentiated thyroid carcinoma. The increased thyroglobulin antibody following immunoglobulin is not a false test result but rather a measurement of the antibody from the pooled human product. This result should not be misinterpreted as disease progression, out of context, in the follow-up of thyroid cancer. Waiting 50 to 60 days for reassessment of thyroglobulin antibody prior to pursuing aggressive cancer-seeking imaging could be considered in the context of a rise in thyroglobulin antibody following immunoglobulin administration.
